# Selected amino acid mutations in HIV-1 B subtype gp41 are Associated with Specific gp120_V3 _signatures in the regulation of Co-Receptor usage

**DOI:** 10.1186/1742-4690-8-33

**Published:** 2011-05-12

**Authors:** Salvatore Dimonte, Fabio Mercurio, Valentina Svicher, Roberta D'Arrigo, Carlo-Federico Perno, Francesca Ceccherini-Silberstein

**Affiliations:** 1 1 University of Rome Tor Vergata, Via Montpellier 1, Rome, Italy; 2 National Institute of Infectious Diseases (INMI) L. Spallanzani, Rome, Italy

## Abstract

**Background:**

The third variable loop (V3) of the HIV-1 gp120 surface protein is a major determinant of cellular co-receptor binding. However, HIV-1 can also modulate its tropism through other regions in gp120, such as V1, V2 and C4 regions, as well as in the gp41 protein. Moreover, specific changes in gp41 are likely to be responsible for of damage in gp120-CCR5 interactions, resulting in potential resistance to CCR5 inhibitors.

In order to genetically characterize the two envelope viral proteins in terms of co-receptor usage, we have analyzed 526 full-length *env *sequences derived from HIV-1 subtype-B infected individuals, from our and public (Los Alamos) databases. The co-receptor usage was predicted by the analysis of V3 sequences using Geno2Pheno (G2P) algorithm. The binomial correlation phi coefficient was used to assess covariation among gp120_V3 _and gp41 mutations; subsequently the average linkage hierarchical agglomerative clustering was performed.

**Results:**

According to G2P false positive rate (FPR) values, among 526 env-sequences analyzed, we further characterized 196 sequences: 105 with FPR <5% and 91 with FPR >70%, for X4-using and R5-using viruses, respectively.

Beyond the classical signatures at 11/25 V3 positions (S11S and E25D, R5-tropic viruses; S11KR and E25KRQ, X4-tropic viruses), other specific V3 and gp41 mutations were found statistically associated with the co-receptor usage. Almost all of these specific gp41 positions are exposed on the surface of the glycoprotein. By the covariation analysis, we found several statistically significant associations between V3 and gp41 mutations, especially in the context of CXCR4 viruses. The topology of the dendrogram showed the existence of a cluster associated with R5-usage involving E25D_V3_, S11S_V3_, T22A_V3_, S129DQ_gp41 _and A96N_gp41 _signatures (bootstrap = 0.88). Conversely, a large cluster was found associated with X4-usage involving T8I_V3_, S11KR_V3_, F20IVY_V3_, G24EKR_V3_, E25KR_V3_, Q32KR_V3_, A30T_gp41_, A189S_gp41_, N195K_gp41 _and L210P_gp41 _mutations (bootstrap = 0.84).

**Conclusions:**

Our results show that gp120_V3 _and several specific amino acid changes in gp41 are associated together with CXCR4 and/or CCR5 usage. These findings implement previous observations that determinants of tropism may reside outside the V3-loop, even in the gp41. Further studies will be needed to confirm the degree to which these gp41 mutations contribute directly to co-receptor use.

## Background

Human immunodeficiency virus type 1 (HIV-1) entry into the host cell is mediated by the viral mature envelope (*env*) glycoproteins, gp120 and gp41, that constitute a trimeric complex anchored on the virion surface by the membrane-spanning segments of gp41 [1-4]. The gp120 exterior glycoprotein is retained on the trimer via labile, noncovalent interactions with the gp41 ectodomain [5], and it must be flexible to allow correct conformational modifications. The initial binding of gp120 to the cellular CD4 receptor indeed triggers conformational changes in gp120 that promote its following interaction with one of the chemokine co-receptors, usually CCR5 or CXCR4 [6-13]. This binding also induces the arrest of the transmembrane gp41 transitions at a prehairpin intermediate stage that leads to the insertion of the fusion peptide into the target cell membrane and ultimately to virus-cell fusion activity [14,15]. Multiple intermolecular contacts are required to maintain trimer integrity in gp120: the C1 and C5 region in gp120 are thought to be a provider to the gp120/gp41 interface and to the disulfide bond loop region of gp41, respectively [5,16-18].

HIV-1 strains can be phenotypically classified according to the virus' ability to use the CCR5 and/or CXCR4 co-receptor. Pure R5-tropic and pure X4-tropic viruses can use only the CCR5 and CXCR4 co-receptors to enter the target cell respectively, while the dual-tropic virus can use both co-receptors [19-23]. The binding to the chemokine receptor is based upon the presence of selected amino acids in gp120 (specifically within the V3 loop, but also in other regions), providing greater affinity to CCR5 or CXCR4, and therefore the viral tropism [24-32].

It has been shown that R5-tropic viruses are generally responsible for the establishment of the initial infection, and they predominate in the majority of drug-naïve patients (prevalence, > 80%) [33-36]. However, in roughly 50% of all infected individuals, the virus changes its chemokine receptor usage during the progression of HIV-1 infection, due to the appearance of dual/mixed viruses [37-44]. Conversely, pure X4-tropic viruses are rare and occur in less than 1% of treatment-naïve patients and less than 5% of treated individuals, even at very late stages of the disease [33-36,45].

Based on the V3 location of the main genetic co-receptor usage determinants, the genotypic approaches for the tropism determination are so far based on sequencing and analyzing the V3 loop of gp120 with different algorithms available online [46,47].

However, emerging data clearly indicate the involvement of other gp120 regions in co-receptor binding, beyond the V3 loop (as V1, V2, and C4), and even that of the gp41 transmembrane protein [48-55]. Interestingly, recent studies have also shown that several mutations in gp41 were found to be significantly associated with co-receptor usage [48,54,56,57].

Therefore, due to the above mentioned reasons, the present investigation aims to genetically characterize HIV-1 B-subtype *env *sequences in terms of co-receptor usage and to define the association of mutations within the gp120 V3-region and the gp41 protein according to CCR5 and/or CXCR4 usage. For this purpose, we analyzed 526 HIV-1 subtype-B *env *sequences, only viral isolates from single patient, mostly retrieved from the Los Alamos database.

## Methods

### Sequence analysis

The analysis included 526 HIV-1 subtype-B *env *full-length sequences, partially retrieved from our database (from 33 HIV-positive patients receiving highly active antiretroviral therapy), and the majority from the Los Alamos database [58]from 493 infected individuals at all stages of infection, with one isolate per single patient [58]. Sequences available with pure phenotype and/or co-receptor determinations have been considered, while molecular clone and dual-mix viruses have not been used. Published *env *consensus sequences of pure HIV-1 (A, B, C, D, F1, F2, G, H, J, and K) were used as reference for each subtypes [58], and multiple sequence alignments of V3 and gp41 segments were performed by using ClustalX [59] and were manually edited with the Bioedit software [60].

### V3 and gp41 sequencing

The sequencing of the V3 gp120 region and the entire gp41 was performed on 33 plasma samples, as described elsewhere [61,62]. In brief, for gp41 sequencing, RNA was extracted, retrotranscribed, and amplified by use of 2 different sequence-specific primers. Gp41-amplified products were full-length sequenced in sense and antisense orientations by use of 8 different overlapping sequence specific primers for an automated sequencer (ABI 3100; Applied Biosystems). Sequences with a mixture of wild-type and mutant residues at single positions were determined to have the mutant(s) at that position. Nucleotide sequences were previously submitted to Genbank [63].

For the sequencing of gp120 V3-domain, HIV-1 RNA was extracted, the V3-containing region of the *env *gene was then reverse-transcribed and amplified using the forward primer V3S2 5' CAGCACAGTACAATGTACACA 3' (nucleotide [nt]: 630-650 of HIV-1 HxB2 gp120 *env *gene) and the reverse primer V3AS5 5' CTTCTCCAATTGTCCCTCA 3' (nt: 1292-1310). The conditions for reverse transcription and amplification were: one cycle at 50°C for 30 min, one cycle 94°C for 2 min, 40 cycles (94°C 30 s, 52°C 30 s, 72°C 40 s), and a final step at 72°C for 10 min, using the following reaction mix: 25 μl of RNA template, 8 μl of 5 mM Mg^++^, 3 μl of Dnase Rnase free water, 0.75 μl of each primer at a concentration of 10 μM, 1 μl of Rnase out (40 U/μl), 1.5 μl of RT/Taq, 1 μl of dNTPs at a concentration of 10 mM for a total of 40 μl.

PCR-products were then sequenced by using the BigDye terminator v.3.1 cycle sequencing kit (Applied-Biosystems), and an automated sequencer (ABI-3100). Four different overlapping sequence-specific primers were used to ensure the coverage of the V3-sequence by at least two sequence segments. The sequencing conditions were: one cycle 96°C 3 min, 25 cycles (96°C 30 s, 50°C 10 s, 60°C 4 min) and the following primers were used: V3S6 5' CTGTTAAATGGCAGTCTAGC 3', V3S5 5' GTTAAATGGCAGTCTAGCAG 3', V3AS1 5' GAAAAATTCCCCTCCACAATT 3' and V3AS3bis 5' CAATTTCTGGGTCCCCTC 3'.

Subtypes were assessed by the construction of phylogenetic trees generated with the Kimura 2-parameter model. The statistical robustness within each phylogenetic tree was confirmed with a bootstrap analysis using 1000 replicates.

### Tropism prediction

Within all 526 gp160-sequences, the V3 region was extrapolated and submitted for tropism prediction to Geno2Pheno algorithm. Geno2Pheno [46] is a bioinformatics tool based on support vector machines. Beyond tropism prediction, it assigns to each V3 sequence a score, called false positive rate (FPR), ranging from 0% to 100%, which represents the probability for a sequence to belong to an R5-virus. According to FPR values, we selected sequences with FPR < 5% (indicating a strong X4 prediction) and sequences with FPR > 70% (indicating a strong R5 prediction) for X4-tropic and R5-tropic viruses, respectively. These sequences, together with the related gp41sequences, were then used for the entire study.

### Statistical analysis

To analyze gp41 and V3 mutations, we calculated the frequency of all mutations in the 345 gp41 amino acids and 35 V3 amino acids, using the *env *selected sequences. Fisher exact tests were used to determine whether the differences in frequency between the 2 groups of patients were statistically significant (sequences with strong R5 and X4 prediction, respectively).

The Benjamini-Hochberg method has been used to identify results that were statistically significant in the presence of multiple-hypothesis testing [64]. A false discovery rate of 0.05 was used to determine statistical significance.

To identify significant patterns of pairwise associations between V3 and gp41 mutations, we calculated the *φ *coefficient and its statistical significance for each pair of mutations. A positive and statistically significant correlation between mutations at two specific positions (0 <*φ *< 1; *P *≤ 0.05) indicates that the latter mutates in a correlated manner in order to confer an advantage in terms of co-receptor selection and that the co-occurrence of these mutations is not due to chance. Moreover, to analyze the covariation structure of mutations in more detail, we performed average linkage hierarchical agglomerative clustering, as described elsewhere [63,65]. Mann-Whitney *U *tests have been used to assess statistically significant differences among all the pairwise mutations associated. Statistical tests have been corrected for multiple-hypothesis testing by using the Benjamini-Hochberg method at a false discovery rate of 0.05 [64].

## Results and Discussion

### Prevalence of mutations

The study included 526 HIV-1 subtype-B *env *sequences, with the majority retrieved from the Los Alamos database. The V3 region was extrapolated from these gp160-sequences and submitted to the Geno2Pheno algorithm for tropism prediction.

Based on the FPR values, we selected 105 V3 sequences with FPR < 5% and 91 sequences with FPR > 70%, for their X4-using and R5-using co-receptor, respectively. These 196 sequences, together with the related gp41sequences, were then used for the rest of the study.

**Figure 1:**
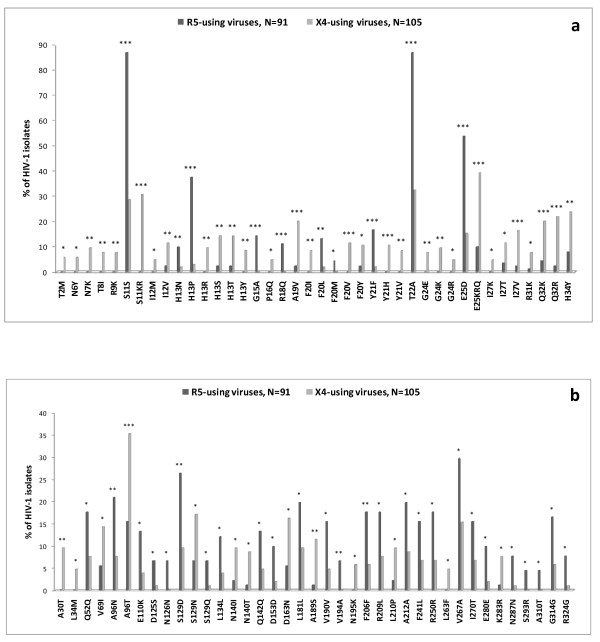
**Frequencies of HIV-1 gp120_V3 _and gp41 mutations**. Frequencies of gp120_V3 _(panel "a") and gp41 (panel "b") mutations in HIV-1 R5-tropic isolates with FPR > 70% by Geno2Pheno-algorithm prediction (dark grey) and HIV-1 X4-tropic isolates with FPR < 5% by Geno2Pheno-algorithm prediction (light grey). Statistically significant differences were assessed by chi-square tests of independence. *P *values were significant at a false-discovery rate of 0.05 following correction for multiple tests. *, *P *< 0.05; **, *P *≤ 0.01; ***, *P *≤ 0.001.

As a first analysis, we confirmed in our dataset that the classical V3 positions 11 and 25 (consistent with previous observations [66-68]), wild-type amino acid at position 11, S11S, and E25D mutation were significantly associated with R5-tropic viruses, while mutations S11KR and E25KRQ were significantly associated with CXCR4 co-receptor usage (Figure 1a).

Since networks of V3 mutations are variable and complex, positions 11 and 25 are not sufficient to provide a full understanding of the mechanisms underlying different co-receptor usage. For example, it has been demonstrated that CCR5 interacts with the conserved V3 region encompassing the residues 4 to 7 (P4-N5-N6-N7) and the binding of this co-receptor is blocked when N7 is replaced by charged amino acid [30]. In our dataset, the mutation N7K has been found only in X4-predicted viruses (prevalence 9.5%; *P *= 0.002) (Figure 1a).

By evaluating the V3 loop sequence, we have identified 9 V3 mutations whose prevalence was significantly higher in the R5-predicted viruses than in the X4-predicted viruses (*P *< 0.05) (Figure 1a). Seven of them had a prevalence > 10% in R5-predicted viruses (the known E25D, and H13P, G15A, R18Q, F20L, Y21F and T22A). We also identified 33 mutations whose prevalence was significantly higher in X4- than in R5-viruses, suggesting their association with CXCR4-usage (*P *< 0.05). Among them, 17 had a prevalence > 10% in X4-predicted viruses (the known S11KR and E25KRQ, and I12V, H13ST, A19V, F20VY, Y21H, I27TV, Q32KR, H34Y), suggesting that within the V3 region, many more mutations are associated with CXCR4 usage (Figure 1a).

Interestingly, the majority of these V3 mutations found associated with the co-receptor usage were also recently found by our group as being involved in mechanisms underlying different co-receptor usage, using a completely different approach and dataset of isolates [68].

In addition, it is important to note that the selected dataset of sequences used in this study is small compared to the total number of sequences available in the Los Alamos database; we also analyzed a different dataset of sequences with known phenotyping determination, composed by 326 and 91 V3-sequences (one HIV-1 B-subtype sequence/patient), with non-syncytium-inducing (NSI)- and syncytium-inducing (SI)-information, respectively.

Almost all statistically significant associations among V3 mutations and tropism found previously in the study were confirmed with this new analysis. The classical R5-tropic determinants S11S and E25D were found with high prevalence in NSI-sequences (73.6% and 64.1%, respectively, versus 34% and 11%, respectively, in SI-sequences; P < 0.05), while the classical X4-tropic mutations S11KR and E25KRQ were found with high prevalence in SI-sequences (40.6% and 51.6%, respectively, versus 2% and 11%, respectively, in NSI-sequences; P < 0.05). Moreover, the novel identified V3 mutations T22A in the R5-predicted viruses, and I12V, A19V, Y21H and H34Y in the X4-predicted viruses were also confirmed (P < 0.05).

The high variability of the V3 loop found in our study should not be surprising, since positive selection has been implicated in the maintenance of such diversity, in individuals as well as at the population level and in co-receptor selection [68-72]. It is likely that the principal driving force in the evolution of the V3 region of HIV-1 is the cell receptor usage, the escape from host immune response, or a combination of the two [73,74].

By analyzing the gp41 sequences, we found 35 out of 345 gp41 positions significantly associated with different co-receptor usage (*P *< 0.05) (Figure 1b). In particular, we identified 13 gp41 mutations whose prevalence was significantly higher in R5-using than in X4-using viruses: 7 of them had a prevalence > 10% in R5-predicted viruses (A69N, E110K, S129D, R209L, F241L, V267A, and I270T). Beyond these mutations, the wild type amino acid at 13 gp41 positions were also significantly associated with the R5-prediction (Q52Q, N126N, L134L, Q142Q, D153D, L181L, V190V, F206F, A212A, R250R, E280E, N287N and G314G) (Figure 1b).

Conversely, we identified 13 mutations whose prevalence was significantly higher in X4- than in R5-viruses, suggesting their association with the CXCR4-usage. Among them, 5 mutations had a prevalence > 10% in X4-predicted viruses (V69I, A96T, S129N, D163N and A189S) (Figure 1b).

Several gp41 residues associated with different co-receptor-usage reside within the Heptad Repeat 1 and 2 (HR1 and HR2) (A30, L34, Q52, D125, N126, S129, L134, N140, N141 and Q142), in the cluster I epitope transiently exposed during fusion (V69), and in the tryptophan-rich membrane-proximal external region (MPER) (D153 and D163). All these positions are localized in gp41 ectodomain known to be immunodominant and to induce high-titer antibodies in the majority of HIV-1-infected individuals [75-81]. The fact that all these mutations are localized in the extracellular domain of gp41 is consistent with the idea that gp41 may act as a scaffold in order to maintain the stability of the gp120/gp41 complex, and therefore finally influencing the viral tropism as well, directly or indirectly.

### Association among mutations

By the analysis of associations between mutations, for the first time we found specific and statistically-significant correlations between V3 and gp41 mutations. In particular, several associations among mutations were associated with the CXCR4 prediction. An exception was represented by the A96N_gp41 _mutation that was positively correlated with T22A_V3 _(*φ *= 0.22; *P *= 0.030; both associated with CCR5-usage) and negatively correlated with the known S11KR mutations (*φ *= -0.17; *P *= 0.018). The A96N_gp41 _mutation is specifically localized in gp41 ectodomain and in particular within the cluster-I, that is a gp41 immunodominant loop involved in the interactions with gp120 [16,18,82-85].

Similarly, S129DQ_gp41_, associated with CCR5-usage and localized in the gp41 HR2 domain, established negative correlation with the S11KR, strongly associated with CXCR4-usage, (*φ *= -0.21; *P *= 0.041) (Table 1). Notably, antibodies directed to the HR1/HR2 complex exist in the sera of HIV-1-infected individuals and this highlights the immunogenic character of the complex [75,86,87].

**Table 1:** Novel gp41 mutations significantly associated with gp120_V3 _mutations

gp41 mutations	**Frequency no. (%) of isolates**^**a**^	**Frequency % in X4-tropic viruses**^**b**^	Correlated mutations	**Frequency no. (%) of isolates**^**a**^	**Covariation frequency no. (%) of isolates**^**c**^	***φ***^**d**^	***P ***^**e**^
**A30T _gp41_**	10 (5.1)	100	**F20IVY _v3_**	34 (17.3)	8 (80.0)	0.38	0.001
			**E25KQR _v3_**	62 (31.6)	9 (90.0)	0.29	0.006
			**S11S _v3_**	109 (55.6)	0 (0)	-0.26	0.009

**L34M _gp41_**	5 (2.5)	100	**N7KTY _v3_**	15 (7.6)	3 (60.0)	0.32	0.055

**A96N _gp41_**	27 (13.8)	29.6	**T22A _v3_**	113 (57.6)	23 (85.2)	0.22	0.03
			**S11KR _v3_**	51 (26.0)	3 (11.1)	-0.17	0.018

**A96T _gp41_**	51 (26.0)	72.5	**N140IT _gp41_**	22 (11.2)	12 (23.5)	0.23	0.054
			**T22A _v3_**	113 (57.6)	19 (37.2)	-0.24	0.022

**S129DQ _gp41_**	43 (20.9)	26.8	**S11KR _v3_**	51 (26.0)	4 (9.8)	-0.21	0.041

**S129N _gp41_**	24 (12.2)	75	**I12MV _v3_**	19 (9.7)	7 (29.2)	0.26	0.041

**N140IT _gp41_**	22 (11.2)	86.4	**N7KTY _v3_**	15 (7.6)	6 (27.3)	0.26	0.046
			**A96T _gp41_**	51 (26.0)	12 (54.5)	0.23	0.054
			**S11S _v3_**	109 (55.6)	5 (22.7)	-0.24	0.028

**A189S _gp41_**	13 (6.6)	92.3	**Q32KR _v3_**	50 (25.5)	9 (69.2)	0.27	0.021

**N195K _gp41_**	6 (3.1)	100	**T8I _v3_**	8 (4.1)	3 (50.0)	0.41	0.022
			**S11KR _v3_**	51 (26.0)	6 (100)	0.16	0.041

**L210P _gp41_**	12 (6.1)	83.3	**G24EKR _v3_**	23 (11.7)	6 (50.0)	0.31	0.019

^a ^Frequency was determined in 196 isolates from HIV-1 infected patients having FPR < 5% and FPR > 70%, using the Geno2Pheno algorithm.

^b ^Frequency was determined in 105 HIV-1 isolates reported as X4-tropic at genotypic test (FPR < 5%).

^c ^Percentages were calculated in patients with each specific gp41 mutation.

^d ^Positive and negative correlations with *φ *> 0.15 and *φ *< -0.15, respectively, are shown.

^e ^*P *values significant (*P *≤ 0.05) after correction for multiple hypothesis testing [65].

Regarding the positive correlations between V3 and gp41 mutations associated with CXCR4-usage, several were localized in the gp41 ectodomain (Table 1). In particular, a strong correlation was observed for A30T_gp41_with either F20IVY_V3 _(*φ *= 0.38; *P *= 0.001) or E25KRQ_V3 _(*φ *= 0.29; *P *= 0.006) (Table 1). Of note, F20IVY_V3 _and E25KRQ_V3 _were found in 80% and 90% of patients with A30T_gp41 _respectively, thus further supporting that these mutations are highly correlated with each other. Another positive correlation was observed for L34M_gp41 _with N7KTY_V3 _(Table 1).

Interestingly, both A30T_gp41 _and L34M_gp41 _were also found recently associated phenotypically with CXCR4 usage [54,56,57]. Specifically, evaluating the available gp41 sequence data from samples submitted for co-receptor tropism testing by Trofile™, a CLIA-validated cell-based recombinant virus assay, Stawiski et colleagues have observed 26 gp41 mutations associated with CXCR4-use (Dual Mix/CXCR4), with the majority being on the extracellular region [56].

A30T_gp41 _and L34M_gp41 _are located in a specific region of HR1 involved in a direct interaction with gp120 [88]. In addition, the presence of A30T_gp41 _and L34M_gp41 _was observed in CXCR4-using isolates characterized by a high infectivity and/or replication capacity in CXCR4-expressing cells, thus supporting their involvement in the mechanism underlying CXCR4 usage [56,89,90]. Overall, this supports the role of these two mutations in the stabilization of non-covalently complex gp120/gp41, and/or in viral receptor attachment and membrane fusion.

Of note, we also found positive correlations between V3 mutations and gp41 mutations localized in the transmembrane domain or in the cytoplasmic tail of gp41. This is the case of A189S_gp41_, localized in gp41 transmembrane domain, which correlated with Q32KR_V3 _(*φ *= 0.27; *P *= 0.021). Both mutations were found positively associated with the CXCR4 prediction. Moreover, it has already been noted that Q32KR_V3 _could determine a reduction of gp120 binding affinity for the CCR5 N-terminus, and this reduction is even stronger than that observed when positive charges are present at the classical V3 positions 11 and 25 [68].

Similarly, L210P_gp41_, localized before the Kennedy sequence (that is a loop of the C-terminal tail of gp41 which is supposed to be exposed on the viral surface [91]), showed a strong correlation with G24EKR_V3 _(*φ *= 0.31; *P *= 0.019).

**Figure 2:**
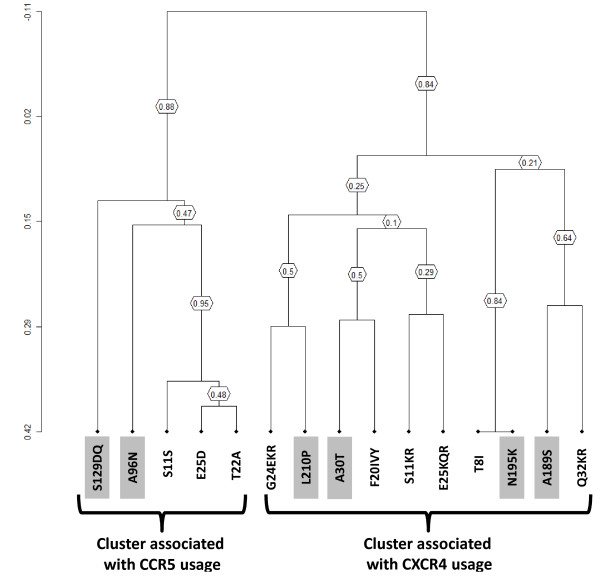
**Clusters of correlated mutations**. Dendrogram obtained from average linkage hierarchical agglomerative clustering, showing significant clusters involving V3 and gp41 (gray box) mutations. The length of branches reflects distances between mutations in the original distance matrix. Boostrap values, indicating the significance of clusters, are reported in the boxes. The analysis was performed in sequences derived from 196 patients, 91 reported as R5-tropic and 105 reported as X4-tropic at genotypic test.

The correlation between V3 and gp41 mutations was also confirmed by hierarchical clustering analysis. In particular, the topology of the dendrogram suggests the existence of a cluster associated with R5-usage and involving S11S, E25D, and T22A in the V3 and A96N and S129DQ in gp41 (bootstrap = 0.88) (Figure 2). Conversely, a large cluster was found associated with X4-usage. This involves the V3 mutations T8I, S11KR, F20IVY, G24EKR, E25KQR, Q32KR along with the gp41 mutations A30T, A189S, N195K, L210P (bootstrap = 0.84) (Figure 2).

Overall, our results suggest that specific additional gp41 mutations could be taken into account in order to implement the genotypic prediction algorithms currently in common use, as already demonstrated by Thielen and colleagues, who observed an improvement (albeit marginal) of CXCR4 co-receptor usage prediction [57]. In this work, it has been shown that mutations at N-terminus of gp41, such as A30T and L34M, are strongly associated with co-receptor phenotype in two independent datasets (444 and 1916 patients screened, respectively). The authors affirm that this region could theoretically be used to predict co-receptor use, alone or in combination with the V3 region. In our study, these 2 mutations, A30T and L34M, were both 100% associated to CXCR4-tropic viruses (Table 1).

It is conceivable that even mutations in gp41 may modulate co-receptor specificity and facilitate efficient CXCR4-mediated entry. This is consistent with other observations that showed that determinants of CXCR4 use in a set of dual-tropic *env *sequences, with V3 sequences identical to those of R5-tropic clones, mapped to the gp41 glycoprotein. Indeed, Huang et colleagues have shown that mutations in the fusion peptide and cytoplasmic tail of gp41 contribute to CXCR4 use by a dual-tropic clone, while a single G515V mutation (according to HXB2 gp140 numbering) in gp41-fusion-peptide of another dual-tropic clone was sufficient to confer CXCR4 use to the R5-tropic original clone [48]. Similarly, the same authors reported previously that for HIV-1 subtype-D the V3 loop sequence of dual-tropic clones was identical to those of co-circulating R5-tropic clones, indicating the presence of CXCR4 tropism determinants also in domains different from V3 [41]. Interestingly, the threonine in position 96 that we find mutated in 72.5% of our viral X4-tropic B-subtype sequences (A96T_gp41_) and negatively correlated with the R5-determinant T22A_V3_, is the wild-type amino acid of gp41 in HIV-1 consensus sequence of subtype D viruses.

Based on crystal structures of HIV-1 gp41 so far available [92-95], the positions A30, L34, A96, S129 and N140 are all exposed on the surface of the glycoprotein (in HR1 or HR2 domains). Similarly, position L210 too, being near the epitopes for neutralizing antibodies, is presumably exposed on the surface glycoprotein [91]. Differently, the position of gp41 N195 seems to be located at the end of the classical single membrane spanning domain (172-198 amino acids), recently proposed to shuttle between two different conformations during the fusion process [96]. The same residue, based on another work [91], is part of an external loop of gp41 in an alternative membrane-spanning model, suggesting its alternating intra- and extra-membrane localization.

Consequently, we could speculate that gp41 A30T, L34M, A96NT, S129DQN, N140IT, N195K and L210P mutations may act together (directly or indirectly) with specific V3 signatures, via allosteric effects on the gp120/gp41 complex. This may allow the best conformational structural plasticity of gp41 and gp120 for their appropriate and specific binding to the cellular receptors and co-receptors. To support this hypothesis, the x-ray crystal structures of CD4-bound HIV-1 gp120 have revealed that the gp120 "core" consists of a gp41-interactive inner domain, a surface-exposed and heavily glycosylated outer domain and a conformationally flexible bridging sheet [14,30,97]. In addition, recent studies showed that in CD4-bound state two potentially flexible topological layers in the gp120 inner domain apparently contribute to the noncovalent association of gp120 with gp41 [98] and insertions in V3 or polar substitutions in a conserved hydrophobic patch near the V3 of gp120 resulting in decreased gp120/gp41 association and decreased chemokine receptor binding [99].

With regard to the gp120-CD4 binding, it was found that the resulting conformational modifications protrude the V3 flexible loop to interact with the cellular co-receptor [29,97]. Interestingly, monoclonal antibodies directed against the D19 epitope within the V3 region had a neutralizing function only for the X4-tropic viruses, regardless of the presence of sCD4, while for R5 isolates only upon addition of sCD4 [100]. Consequently, the inaccessibility of this antibody to R5-tropic viruses in the absence of sCD4 might indicate that there are significant V3 loop conformational differences between these two viral variants [101], but also that specific interactions occurring in the gp120/gp41 complex may participate in the HIV-1 co-receptor usage and neutralization sensitivity.

Finally, we should mention that Anastassopoulou et colleagues have shown that viruses resistant to the small molecule CCR5 inhibitor, vicriviroc, can be caused by 3 conservative changes in the fusion peptide of HIV-1 gp41 [102], and similarly Pfaff *et al.*, very recently, found the involvement of gp120 and gp41 mutations in modulating the magnitude of drug resistance to another small CCR5 antagonist, aplaviroc [103]. Overall, these studies, which focus on changes toward resistances without assessing the issue of tropism-switch, are complementary to our results.

## Conclusions

In this study, we found that specific gp41 mutations are significantly associated with different co-receptor usage and with specific V3 mutations, thus providing new information that could be taken into account for improving co-receptor usage prediction. These findings implement previous observations that determinants of tropism may reside outside the V3 loop, even in the gp41 transmembrane protein. It is possible that the gp120/gp41 complex may become structurally or functionally involved at different stages during virus-cell entry and fusion. Probably, the associations among V3 and gp41 mutations may also have an impact on the HIV pathogenesis, it is known that CXCR4 phenotype has been associated with progression and increased severity of HIV disease, and several gp41 mutations are associated with viral fitness and cytopatic effects. Additional studies are needed to confirm the degree to which these gp41 mutations contribute directly to co-receptor use and to establish the specific and precise utility of this information.

## Competing interests

The authors declare that they have no competing interests.

## Authors' contributions

SD and FM participated in the design of the study and performed the tropism prediction and statistical analysis. SD drafted the manuscript. RD was responsible for HIV-1 sequencing. VS, FCS and CFP participated in the study design and coordination and helped on writing the manuscript. All authors read and approved the final manuscript.
